# Cell shape, and not 2D migration, predicts extracellular matrix-driven 3D cell invasion in breast cancer

**DOI:** 10.1063/1.5143779

**Published:** 2020-05-05

**Authors:** Janani P. Baskaran, Anna Weldy, Justinne Guarin, Gabrielle Munoz, Polina H. Shpilker, Michael Kotlik, Nandita Subbiah, Andrew Wishart, Yifan Peng, Miles A. Miller, Lenore Cowen, Madeleine J. Oudin

**Affiliations:** 1 Department of Biomedical Engineering, Tufts University, Medford, Massachusetts 02155, USA; 2 Department of Computer Science, Tufts University, Medford, Massachusetts 02155, USA; 3 Center for Systems Biology, Massachusetts General Hospital Research Institute, Boston, Massachusetts 02114, USA

## Abstract

Metastasis, the leading cause of death in cancer patients, requires the invasion of tumor cells through the stroma in response to migratory cues, in part provided by the extracellular matrix (ECM). Recent advances in proteomics have led to the identification of hundreds of ECM proteins, which are more abundant in tumors relative to healthy tissue. Our goal was to develop a pipeline to easily predict which ECM proteins are more likely to have an effect on cancer invasion and metastasis. We evaluated the effect of four ECM proteins upregulated in breast tumor tissue in multiple human breast cancer cell lines in three assays. There was no linear relationship between cell adhesion to ECM proteins and ECM-driven 2D cell migration speed, persistence, or 3D invasion. We then used classifiers and partial-least squares regression analysis to identify which metrics best predicted ECM-driven 2D migration and 3D invasion responses. We find that ECM-driven 2D cell migration speed or persistence did not predict 3D invasion in response to the same cue. However, cell adhesion, and in particular cell elongation and shape irregularity, accurately predicted the magnitude of ECM-driven 2D migration and 3D invasion. Our models successfully predicted the effect of novel ECM proteins in a cell-line specific manner. Overall, our studies identify the cell morphological features that determine 3D invasion responses to individual ECM proteins. This platform will help provide insight into the functional role of ECM proteins abundant in tumor tissue and help prioritize strategies for targeting tumor-ECM interactions to treat metastasis.

## INTRODUCTION

Metastasis, the dissemination of cells from the primary tumor to secondary organs in the body, is the leading cause of death in cancer. Metastasis involves the local invasion of tumor cells into the surrounding tissues, intravasation into the vasculature and lymphatics, and colonization of a distant site. All steps within tumor progression require cell migration—growth, invasion,^1^ and metastatic outgrowth.^2^ Understanding the mechanisms that drive cell migration in cancer is essential to identify strategies to treat cancers more effectively. Within tumors, several chemical and biophysical cues have been shown to promote local invasion.^3^ In particular, the extracellular matrix (ECM), which provides structure and support to our tissues, drives local invasion of tumor cells and metastasis, as well as colonization of secondary sites. For example, the glycoprotein Fibronectin, which is produced by both tumor and stromal compartments in breast tumors,^4^ can drive directional migration of breast cancer cells to drive metastasis.^5^ The optimization of protocols to characterize the ECM of tumors has led to the identification of multiple ECM proteins abundant in tumor tissue that may be involved in promoting metastatic phenotypes.^4,6^ The present study aims to develop a pipeline to easily assess which of these ECM proteins, alone or in combination, are more likely to affect invasion and metastasis, and are therefore better targets as biomarkers or for drug development.

Breast cancer cells sense ECM cues within their environment via cell surface receptors and the extension of actin-rich protrusions such as lamellipodia and filopodia. The activation of downstream signaling pathways and the formation of focal adhesions promote cytoskeletal dynamics, which help the cell propel itself forward, eventually retracting its tail via disassembly of focal adhesions. Cell-ECM interactions and their impact on cell behavior can be studied in different contexts. Cell responses to ECM cues have been measured as alterations in the cell shape following adhesion to a substrate, 2D migration on a substrate, and 3D invasion into a matrix containing the ECM substrate. However, we still do not understand the relationship between adhesion to, 2D migration on, and 3D invasion in a given ECM substrate. Therefore, there is a critical need to create a predictive model to use cell morphology to predict cell invasion responses to ECM cues.

Existing models that predict cell migration have focused on cell morphology or signaling pathways and mostly focused on a single cue. First, cell morphology or shape is commonly used to characterize cellular phenotypes, because it can be easily visualized and quantified using traditional immunostaining and basic microscopy. Epithelial keratocytes from fish skin have been used to generate various models due to their characteristic and homogeneous fan-like shape. Various models have been published linking the cell shape and geometry with cell migration and speed.^7,8^ This has been more challenging for cancer cells given their more complex and heterogeneous cell morphologies. There have been efforts to identify signaling pathways that regulate cell morphology. One study linked breast cancer cell morphology *in vitro* in 3D Matrigel with gene expression to identify dominant genes that are predictive of morphological features.^9^ Quantitative morphological profiling has also been used to evaluate the role of individual genes in regulating the cell shape using genetic screens in drosophila cells, leading to the identification of signaling networks that regulate cell protrusion and adhesion.^10^ In response to Collagen I, 3D cell migration correlated with cell protrusion and not with 2D migration speed or persistence.^11^ However, these studies all focus on a single ECM cue. One study investigated the relationship between membrane protrusion, 2D cell migration, and 3D invasion in response to growth factors, which can promote invasion and metastasis.^12,13^ Meyer *et al.* demonstrated that cell protrusion in response to growth factors was more predictive of 3D invasion, than 2D invasion. However, this study focused on soluble cues, which passively diffuse into cells and signal via receptor tyrosine kinases. There are currently no studies dissecting the relationship between ECM-driven effects on the cell shape, 2D migration, and 3D invasion and predicting mesenchymal 3D cell movement in response to ECM cues.

The goal of this study is to understand how ECM cues in the tumor microenvironment promote invasion and metastasis of cancer cells from the primary tumor. Using classification and regression models trained on cell morphology, 2D migration, and 3D invasion data, we find that the cell shape in response to a particular ECM protein can predict the ability of a cell line to invade through that ECM protein in 3D.

## RESULTS

### ECM impacts breast cancer cell adhesion

We chose to build our model using four ECM proteins known to be abundant in breast tumors: Collagen I, Collagen IV, Fibronectin, and Tenascin C. These components were identified in xenograft 4T1 breast tumors^6^ and in highly metastatic LM2 tumors.^4^ Collagen I and the glycoprotein Fibronectin are two of the most abundant ECM proteins in mammary tumors, and both are known to contribute to breast cancer invasion and metastasis.^5,14^ Another glycoprotein, Tenascin C, has also been shown to contribute to breast cancer metastasis.^15^ Collagen IV is a major component of the basement membrane, which breast cancer cells must break down to invade surrounding tissues.^16,17^ To investigate the effects of these ECM proteins, we used two human triple-negative breast cancer cell lines, MDA-MB-231 and MDA-MB-468. MDA-MB-231 is mesenchymal, with high metastatic potential in mouse models, while MDA-MB-468 is epithelial, with lower metastatic potential.^18,19^

**FIG. 1.**
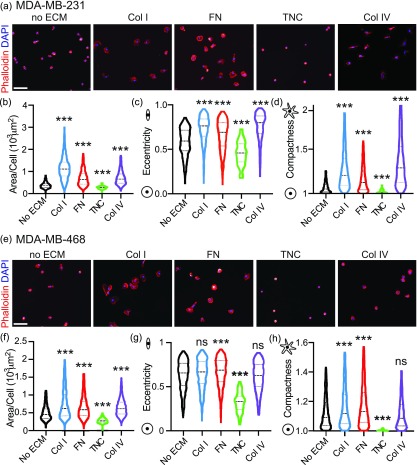
ECM proteins upregulated in breast tumor tissue have distinct cell line-specific effects on tumor cell adhesion. (a) Representative images of MDA-MB-231 cells plated on plastic, Collagen I, Fibronectin, Tenascin C, or Collagen IV for 2 h, fixed and stained with Phalloidin (red) and DAPI (blue). The scale bar is 100 *μ*m. Quantification of cell shape features using Cell Profiler to evaluate effects on MDA-MB-231: (b) area/cell (10^3^ *μ*m^2^), (c) eccentricity, and (d) compactness. Results show entire distribution, no ECM (n = 793 cells), Collagen I (n = 907 cells), Fibronectin (n = 1154 cells), Tenascin C (n = 104 cells), or Collagen IV (n = 766 cells). Significance by one-way ANOVA, ^***^p < 0.005. (e) Representative images of MDA-MB-468 cells plated on plastic, Collagen I, Fibronectin, Tenascin C, or Collagen IV for 2 h, fixed and stained with Phalloidin (red) and DAPI (blue). Scale bar is 100 *μ*m. Quantification of cell shape features using Cell Profiler to evaluate effects on MDA-MB-468: (f) area/cell (10^3^ *μ*m^2^), (g) eccentricity, and (h) compactness. Results show entire distribution, no ECM (n = 802 cells), Collagen I (n = 1016 cells), Fibronectin (n = 945 cells), Tenascin C (n = 101 cells), or Collagen IV (n = 1073 cells). Significance by one-way ANOVA, ^***^p < 0.005, ns is not significant.

First, we performed an adhesion assay, which has been commonly used to study cell-ECM interactions, where cells are plated on a 2D ECM-coated surface and left to adhere for 2 h. The cells are then fixed and immunostained to assess the cell shape. We focused our efforts on the actin cytoskeleton, given that the cell shape is associated with adhesion and cell migration [Fig. 1(a)]. We quantified 11 cell shape parameters via Cell Profiler, including the cell area, radius, feret diameters, perimeter, aspect ratio, eccentricity (elongation), compactness (irregularity), solidity (irregularity), extent (spread), and form factor (circularity). Using these shape parameters, we established effects of all four ECM proteins on the cell shape. Collagen I, Fibronectin, and Collagen IV led to increased cell area, eccentricity, which characterizes cell elongation, and compactness, which quantifies cell shape irregularity. Tenascin C decreased the cell area, eccentricity, and compactness [Figs. 1(b)–1(d)]. While MDA-MB-468 cells had a smaller cell area on average, the effect of individual ECM proteins had similar relative effects on cell morphology [Figs. 1(e)–1(h)]. These data comprehensively characterize the effect of four ECM proteins upregulated in breast tumor tissue on the breast cancer cell shape.

**FIG. 2.**
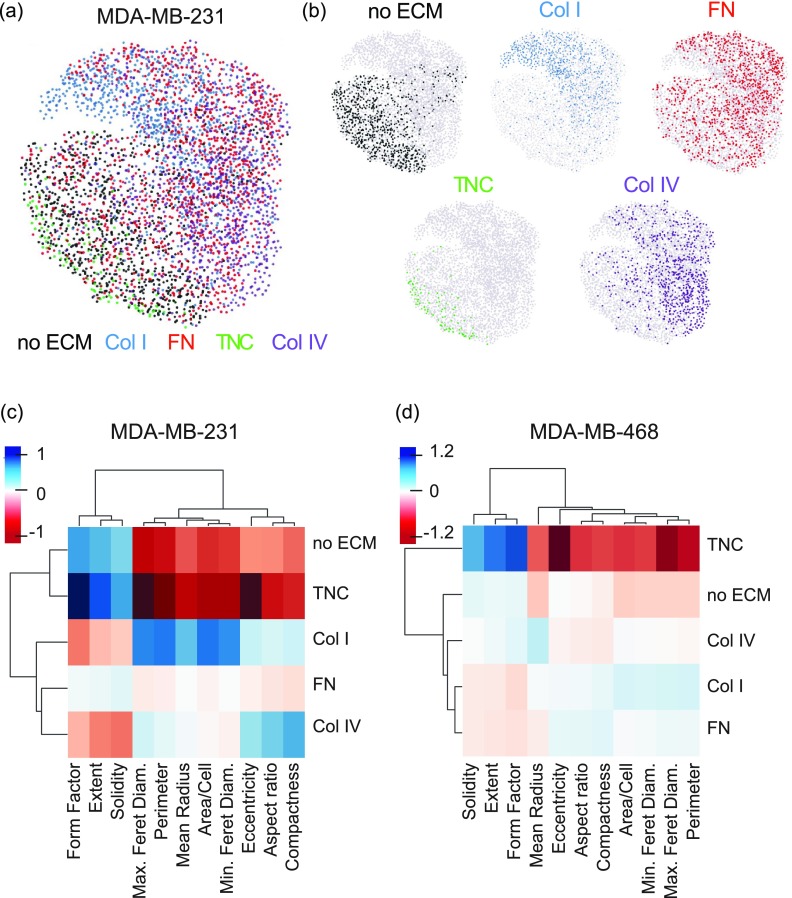
Clustering of adhesion parameters reveals ECM-specific effects on cell shape. (a) Visualization of continuum of cell adhesion of MDA-MB-231 cells on different ECM substrates based on all 11 cell shape parameters with SPRING plots. (b) Plots showing localization of the ECM factor-dependent cell adhesion shape on the combined SPRING plot. Mean centered cell adhesion of MDA-MB-231 (c) and MDA-MB-468 (d) cells plated on plastic, Collagen I, Fibronectin, Tenascin C, or Collagen IV for 2 h. Each cell adhesion parameter and ECM factor are clustered by rank correlation and mean linkage.

To better visualize the effects of ECM proteins on shape parameters, we used SPRING, a pipeline for data filtering, normalization, and visualization using force-directed layouts of k-nearest neighbor algorithms [Fig. 2(a)]. SPRING has been shown to reveal more detailed biological relationships than existing approaches, with plots being more reproducible than those of stochastic visualization methods such as tSNE.^20^ Individual ECMs were mapped onto the individual cells on the SPRING plot [Fig. 2(b)]. For the MDA-MB-231 cells, as was seen in the clustering, cells on Tenascin C and no ECM cluster together. Interestingly, cells on Collagen I are seen as very distinct from cells on no ECM, with little overlap. Cells on Collagen IV are also distinct from cells on no ECM, but are also separate from the cells on Collagen I. Finally, the cells on Fibronectin are homogeneously distributed throughout the cluster. For MDA-MB-468 cells, the distributions of each ECM protein follow similar trends as seen in the clustering [Figs. S1(c) and S1(d)].

Initial analysis of the entire dataset by unsupervised clustering demonstrated that ECM-driven effects on shape parameters cluster into three main groups: one with compactness, aspect ratio, and eccentricity, which quantify how elongated is a cell is, a second group with solidity, form factor and extent, which describe how irregular the shape of the cell is or how protrusive a cell is; and a third group with the feret diameters, radius, perimeter, and cell area, which quantify how large a cell is. The shape parameters for both cell lines clustered in these groups are shown in Figs. S1(a) and S1(b). While the effect of the different ECM proteins on these parameters clustered differently in each cell line, it is clear that Tenascin C shape quantifications are more similar to the no ECM shapes, while Fibronectin and Collagen IV shape characteristics tend to cluster with each other [Figs. 2(c) and 2(d)]. Overall, these clustering methods demonstrate that ECM proteins have distinct effects on the cell shape.

### ECM-driven 2D migration does not correlate with the cell shape

**FIG. 3.**
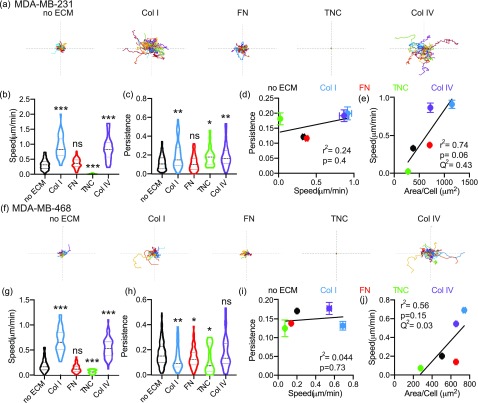
ECM-driven effects on 2D cell migration speed do not correlate with effects on persistence. (a) Representative roseplots for MDA-MB-231 cells plated on glass, Collagen I, Fibronectin, Tenascin C, or Collagen IV for 16 h and imaged every 10 min. Each line represents an individual cell. Axis length is 500 *μ*m. Quantification of cell migration speed (*μ*m/min) (b) and persistence (c). Results show entire distribution, no ECM (n = 128 cells), Collagen I (n = 45 cells), Fibronectin (n = 91 cells), Tenascin C (n = 25 cells), or Collagen IV (n = 39 cells). Correlation between 2D persistence and 2D cell migration speed (d) and between cell area (*μ*m^2^) and 2D cell migration speed (e). Correlation characterized by r^2^, p value, and Q^2^. (f) Representative roseplots for MDA-MB-468 cells plated on glass, Collagen I, Fibronectin, Tenascin C, or Collagen IV for 16 h and imaged every 10 min. Each line represents an individual cell. The axis length is 500 *μ*m. Quantification of cell migration speed (*μ*m/min) (g) and persistence (h). Results show entire distribution, no ECM (n = 220 cells), Collagen I (n = 68 cells), Fibronectin (n = 126 cells), Tenascin C (n = 24 cells), or Collagen IV (n = 63 cells). Correlation between 2D persistence and 2D cell migration speed (i) and between the cell area and 2D cell migration speed (j). Correlation characterized by r^2^, p value, and Q^2^. Significance determined by one-way ANOVA, ^*^p < 0.05, ^**^p < 0.01, ^***^p < 0.005, ns is not significant.

We then investigated the effect of these same ECM cues on 2D cell migration, by evaluating cell migration speed and persistence. Cell speed measures how fast a cell is moving over a given distance, while persistence, the Euclidean distance between start and finish over the total distance traveled, informs whether the cell is moving in a straight line (closer to 1) or taking a more winding path. In MDA-MB-231 cells, we find that Collagen I and Collagen IV increase both cell migration speed and persistence [Figs. 3(a)–3(c)]. Fibronectin has no effect on cell migration speed or persistence; Tenascin C decreases cell migration speed while increasing persistence. Similar results were obtained with the MDA-MB-468 cell line, where Collagen I and Collagen IV increased cell migration speed and persistence, and Tenascin C reduced cell migration speed and persistence [Figs. 3(f)–3(h)]. We find that for all ECM conditions across both cell lines, there is no significant correlation between cell migration speed and persistence at a population and single cell level [Figs. 3(d), 3(i), S2(a), and S2(b)].

To understand whether there is a correlation between cell adhesion and 2D migration, we evaluated how well cell area correlated with cell migration speed and persistence. We found that for both individual cell lines, there was no significant correlation between the cell area and cell migration speed [Figs. 3(e) and 3(j)]. We also find no significant correlation between the cell area and persistence [Figs. S2(c) and S2(d)]. Although the correlation between the cell area and cell migration speed when combining both cell lines is statistically significant, the fit of the linear correlation, R^2^, is low suggesting poor correlation [Fig. S2(e)]. There is no significant correlation between the cell area and persistence when combining the cell lines [Fig. S2(f)]. We also evaluated model prediction accuracy using a leave-one-out cross validation metric, Q^2^, where values lower than 0.5 indicate poor prediction accuracy. All of the correlations investigated had a Q^2^ value below 0.5, suggesting that the cell area alone cannot accurately predict cell migration speed or persistence. Overall, these findings suggest that ECM-driven effects on cell speed and persistence are distinct, and that effects on cell adhesion may not correlate with effects on 2D migration.

### ECM-driven 3D invasion does not correlate with 2D migration or cell shape

**FIG. 4.**
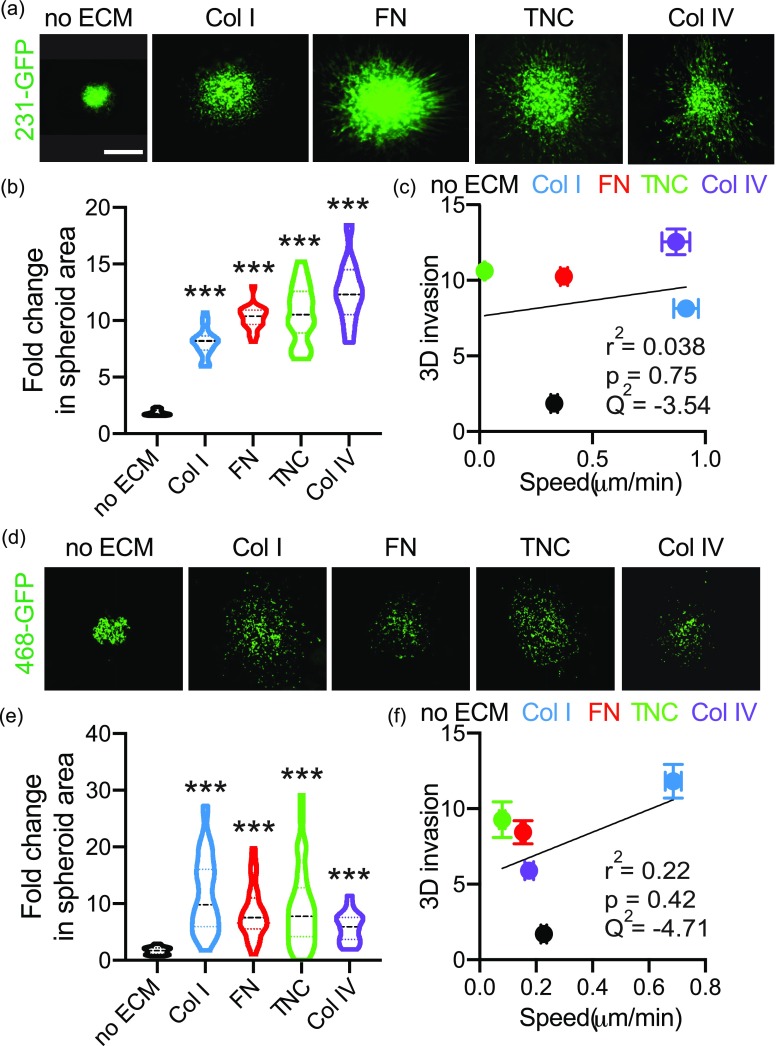
ECM-driven 3D invasion does not correlate with effects on 2D cell migration. (a) Representative images of spheroids made from 231-GFP cells embedded in media, Collagen I, Fibronectin, Tenascin C, or Collagen IV gels for 5 days. The scale bar is 200 *μ*m. (b) Quantification of fold change in 231-GFP spheroid area on day 5 relative to day 1. Data pooled from at least five biological replicates, with three technical triplicate per experiment. ^***^p < 0.001 by one-way ANOVA and Dunn's multiple comparison test. (c) Correlation between mean fold change in spheroid area and 2D cell migration speed for 231-GFP cells. Correlation characterized by r^2^, p value and Q^2^. (d) Representative images of spheroids made from 468-GFP cells embedded in media, Collagen I, Fibronectin, Tenascin C, or Collagen IV gels for 5 days. Scale bar is 200 *μ*m. (e) Quantification of fold change in 468-GFP spheroid area on day 5 relative to day 1. Data pooled from at least four biological replicates, with three technical triplicate per experiment. ^***^p < 0.001 by one-way ANOVA and Dunn's multiple comparison test. (f) Correlation between mean fold change in spheroid area and 2D cell migration speed for 468-GFP. Correlation characterized by r^2^, p value, and Q^2^.

It is well established that 3D invasion is a more physiologically relevant model of *in vivo* cell migration; therefore, we quantified the effect of the individual ECM proteins on 3D invasion in Collagen I gels. We used a spheroid model of 3D invasion, which constitutes microtumors recapitulating various clinically important characteristics like hypoxia, nutrient, and pH gradients and deposition of ECM. All spheroid gels contain Collagen I as a matrix to support spheroid formation, given that it is the most abundant ECM component of breast tissue, and that all ECM proteins present in tumors would be in the presence of Collagen I. We find that all four ECM proteins drive a significant increase in invasion relative to Collagen I only in MDA-MB-231 cells [Figs. 4(a) and 4(b)]. Similarly, the four ECM proteins also increase invasion of MDA-MB-468 cells [Figs. 4(d) and 4(e)], although these cells migrate more individually than the MDA-MB-231 cells.

We then evaluated the correlation between cell adhesion and 3D invasion and between 2D migration and 3D invasion. For both cell lines individually and combined, we do not find a significant correlation between the effects of these proteins on 3D invasion and either 2D migration or cell area [Figs. 4(c), 4(f), and S3]. Additionally, the Q^2^ for each of these models is negative, indicating poor prediction of 3D invasion using cell area, cell migration speed, or persistence. Overall, these findings suggest that ECM-driven effects on 3D invasion may not correlate with effects on the cell area and 2D migration.

### Generation of the classifier-based model suggests that cell adhesion can be used to categorize 2D migration and 3D invasion

To dissect the relationship between ECM-driven cell adhesion, 2D migration, and 3D invasion and develop methods to predict ECM-driven effects on breast cancer cells, we first used machine learning classifier models. Our goal was to evaluate the ability of a learning algorithm to predict 2D migration based on cell adhesion parameters and 3D invasion based on either adhesion parameters or 2D migration. We focused on the AdaBoost classifier, originally developed by Freund and Shapiro in 1995, which is an approach founded on the notion of using a set of weak classifiers and pooling the classification results of such classifiers to produce a provably strong classifier. It is well suited for smaller datasets and also less susceptible to overfitting than other learning algorithms.

**FIG. 5.**
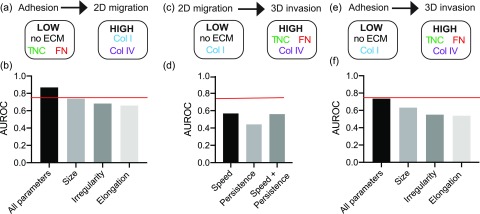
Adhesion classifies ECM-driven 2D migration and 3D invasion. (a) Cell adhesion to predict binary classification of 2D cell migration speed of MDA-MB-231 cells on plastic, Collagen I, Fibronectin, Tenascin C, or Collagen IV. (b) AUROC scores of binary AdaBoost classifier models (a) using all 11 cell shape parameters, cell size parameters (area/cell, perimeter, mean radius, min feret diameter, max feret diameter), cell irregularity parameters (solidity, extent, form factor), and cell elongation parameters (eccentricity, aspect ratio, compactness). (c) 2D cell migration to predict binary classification of mean fold change of spheroid area of 231-GFP cells embedded in media, Collagen I, Fibronectin, Tenascin C, or Collagen IV. (d) AUROC scores of binary classifier models (c) using 2D cell migration (cell migration speed and persistence alone or together). (e) Cell adhesion to predict binary classification of mean fold change of spheroid area of 231-GFP cells embedded in media, Collagen I, Fibronectin, Tenascin C, or Collagen IV. (f) AUROC scores of binary classifier models (e) using all 11 cell shape parameters, cell size parameters, cell irregularity parameters, and cell elongation parameters.

First, we assessed the predictive relationship between cell adhesion and 2D cell migration in response to different ECM proteins. We assigned each ECM protein as either as “low” or “high,” based on its ability to induce 2D cell migration within a cell line. Based on the results in Fig. 3, 2D cell migration speed of MDA-MB-231 cells on no ECM, Fibronectin, and Tenascin C was classified as low [Fig. 5(a)]. The ability of an algorithm to accurately predict whether an ECM protein has a low or high effect is assessed via the Area Under the Curve Receiver Operating Characteristic (AUROC) score, a performance measurement for classification problems. We used AdaBoost to rank single features by their performance on the training set as a one-feature classifier [Fig. S4(a)]. Then we look at ten different AdaBoost classifiers, the first one using just the most informative feature, the second using the five most informative features, and the last one using all 11 features. A grid search of the hyperparameters on the training set showed that it was quite robust to different settings for these classifications (Fig. S4). We chose a combination hyperparameter values with an average AUROC value of 0.75, to prevent overtraining.

Interestingly, using all 11 cell shape parameters were able to predict 2D migration, with an AUROC score higher than 0.80 [Fig. 5(b)]. We then tested whether any of the groups of cell features identified in Fig. 2, cell size, irregularity, and elongation could independently predict 2D migration. We found that the cell size parameters (area/cell, perimeter, mean radius, min, and max feret diameter) could also accurately predict 2D migration speed with AUROC scores of 0.75, while cell elongation and irregularity could not. We found similar results with the MDA-MB-468 cells, where for 2D cell migration, we classified no ECM, Tenascin C and Fibronectin as low, and Collagen I and Collagen IV as high [Fig. S5(a)]. All 11 parameters were able to accurately predict 2D cell migration with an AUROC over 0.75 [Fig. S5(b)]. These data demonstrate that the cell shape of cells adhered to a particular ECM protein is a reliable metric for predicting how this protein will impact 2D migration speed.

Next, we assessed the predictive relationship between cell adhesion or 2D cell migration and 3D invasion in response to different ECM proteins. Based on the results from Fig. 4, 3D invasion of MDA-MB-231 cells embedded in no ECM and Collagen I was classified as low, while 3D invasion in Fibronectin, Tenascin C, and Collagen IV was classified as high [Figs. 5(c) and 5(e)]. We performed a similar hyperparameter grid search to prevent overtraining [Fig. S4(e)]. For both MDA-MB-231 and MDA-MB-468 cells, the AUROC scores for the AdaBoost classifier model are insignificant (around 0.5), suggesting that the classifier was operating with a similar accuracy to that of a random classification assignment [Figs. 5(d) and S5(d)].

Finally, we assessed the predictive relationship between cell adhesion and 3D cell invasion in response to different ECM proteins. We used AdaBoost to rank single features by their performance on the training set as a one-feature classifier [Fig. S4(d)]. Then, we look at ten different AdaBoost classifiers, the first one using just the most informative feature (perimeter), the second using the five most informative features, and the last one using all 11 features. A grid search of the hyperparameters on the training set showed that it was quite robust to different settings for these classifications [Fig. S4(e)]. We chose a combination hyperparameter values with an average AUROC value of 0.75, to prevent overtraining. For both MDA-MB-231 and MDA-MB-468 models, using all shape parameters yielded the highest AUROC scores [Figs. 5(f) and S5(f)]. Models constructed with cell size parameters, cell irregularity parameters, and cell elongation parameters did not have significant AUROC scores. It is notable that the results from classifying with all parameters were all higher than the AUROC scores obtained from just relying on parameters associated with cell size. This demonstrates that cell adhesion can classify 3D cell invasion more accurately than 2D cell migration.

### PLS models suggest that cell adhesion accurately predicts 3D invasion

We then used data-driven modeling to more precisely determine the relationship between ECM-driven cell adhesion, 2D migration, and 3D invasion. First, we used principal components analysis (PCA) to reduce the dimensionality of the cell adhesion dataset. The PCA creates a new set of principal components (PCs), which maximize the covariance captured between the parameters.^21^ Using two principal components, over 80% of the variation in cell adhesion is described, and the distinct effects of each ECM substrate can be identified [Figs. S6(a)–S6(d)]. The PCA shows similar ECM-specific distributions of cell adhesion seen in the SPRING plots, indicating that this data dimensionality reduction method still captures the important ECM-driven trends.

**FIG. 6.**
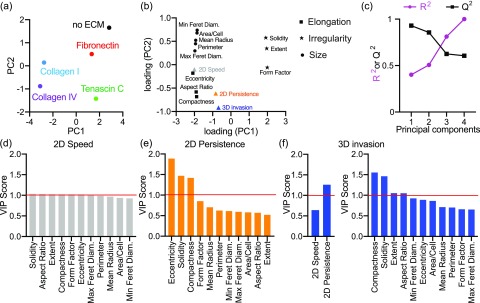
A partial least squares regression model constructed to predict ECM-driven 2D migration and 3D invasion from cell adhesion. (a) Scores plot for PLS model with MDA-MB-231 cells. Principal components reflect covariation between adhesion, 2D migration, and 3D invasion for each cell line on each ECM protein. (b) PLS loading plot for 11 cell adhesion shape parameters and three cell responses. (c) R^2^ and Q^2^ for the PLS model built with increasing numbers of principal components. R^2^ reports model fit, and Q^2^ reports model prediction accuracy using leave-one-out cross validation. Ranked VIP scores for predicting 2D cell migration speed (d), 2D persistence (e), and 3D invasion (f) from cell adhesion or 2D migration. VIP score >1 indicates important parameters to predict the cell invasion response.

Next, we used a partial least squares regression (PLS) to identify covariation between cell adhesion, 2D migration, and 3D invasion. The PLS model reduces the data to a set of principal components (PCs) to optimally describe the proposed relationship between the input, cell adhesion, and the outputs, 2D migration, and 3D invasion.^21^ We constructed the PLS model with MDA-MB-231 cells only (Fig. 6), MDA-MB-468 cells only (Fig. S7), and combining both cell lines (Fig. S8). The scores plot of principal component one (PC1) and PC2 describes how strongly each ECM factor projects on each principal component [Fig. 6(a)]. For example, in MDA-MB-231 cells, Collagen I and Collagen IV project negatively on PC1, whereas no ECM, Fibronectin, and Tenascin C project positively on PC1. Therefore, using both PC1 and PC2, we can distinguish the variation between the effects of different ECM substrates. For the combined model, both PC1 and PC2 are required to describe variation between different cell lines and ECM substrates [Fig. S8(a)]. For no ECM, Fibronectin, and Tenascin C, both cell lines project similarly onto the principal components. However, for Collagen I and Collagen IV, the cell lines project differently onto the principal components.

**FIG. 7.**
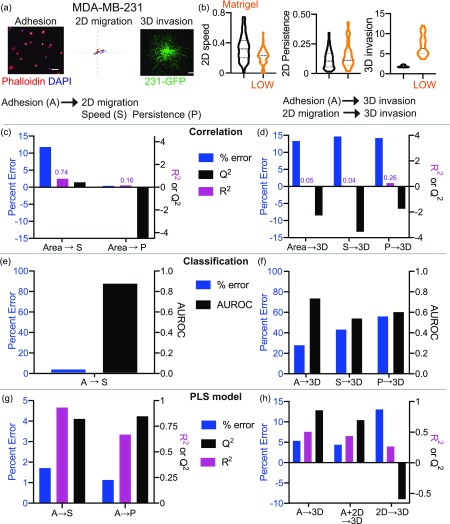
Correlation, classifier, and PLS models accurately predict Matrigel-driven responses in MDA-MB-231 breast cancer cells. (a) Representative cell adhesion, 2D migration, and 3D invasion of MDA-MB-231 cells on or in Matrigel. Representative cell adhesion images show cells fixed and stained with Phalloidin (red) and DAPI (blue) after 2 h on Matrigel. The scale bar is 100 *μ*m. 2D migration roseplots show individual cell tracks on Matrigel for 16 h. The axis length is 500 *μ*m. Representative 3D invasion spheroids are made from 231-GFP cells embedded in Matrigel gels for 5 days. The scale bar is 200 *μ*m. (b) Quantification of cell migration speed, persistence, and 3D invasion of MDA-MB-231 cells in response to no ECM and Matrigel. Prediction of 2D cell migration speed, 2D persistence (c), and 3D invasion (d) of MDA-MB-231 cells on Matrigel based on the simple correlation, assessed by percent error, R^2^ and Q^2^. Accuracy of prediction of 2D cell migration speed (e) and 3D invasion (f) from classifier models determined by percent error and AUROC. Prediction of 2D cell migration speed, 2D persistence (g), and 3D invasion (h) of cells on Matrigel from MDA-MB-231 cells using the PLS model built with two principal components, assessed by percent error, R^2^ and Q^2^.

**FIG. 8.**
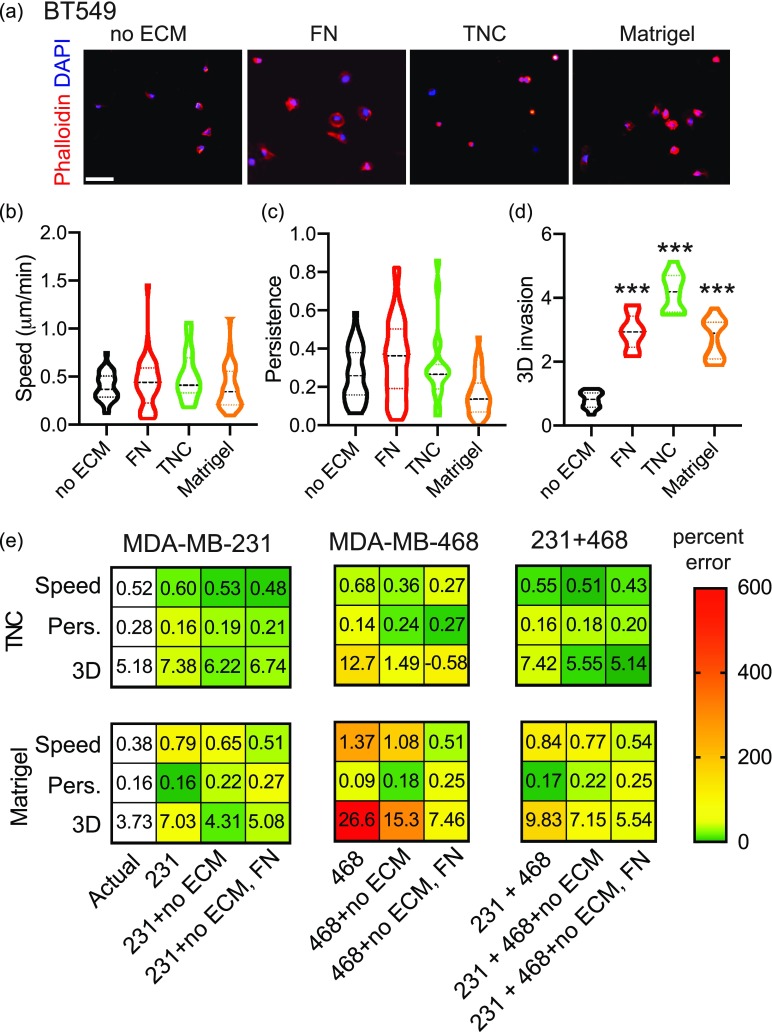
ECM-driven predictions are cell-line specific. (a) Representative cell adhesion images show cells fixed and stained with Phalloidin (red) and DAPI (blue) after 2 h on Matrigel. The scale bar is 100 *μ*m. Quantification of cell migration speed (b) and persistence (c). Data show entire distribution, with no ECM (n = 30), FN (n = 36), TNC (n = 19), Matrigel (n = 36). (d) Quantification of fold change in BT-549-GFP spheroid area on day 5 relative to day 1. Data pooled from 1 biological replicate, with 11 technical triplicate. ^***^p < 0.001 by one-way ANOVA and Dunn's multiple comparison test. (e) Prediction of 2D cell migration speed, 2D persistence, and 3D invasion of BT-549 cells on Tenascin C and Matrigel from MDA-MB-231, MDA-MB-468, and combined PLS models built with two principal components for MDA-MB-231 model and three principal components for MDA-MB-468 and combined cell lines models. Predictions were done without BT-549 data, with the addition of BT-549 no ECM, and with the addition of BT-549 no ECM and Fibronectin. Numbers represent the actual and predicted values for each metric. Colors represent percent error, where green is a low error and red is a high error value, indicated by the color gradient.

To understand the effects of cell adhesion parameters in the model, we projected the loading vectors, which describe how strongly each parameter projects onto each principal component [Fig. 6(b)]. We find that measures of cell size, irregularity and elongation project in distinct clusters, such that cell irregularity projects positively on PC1 and cell size and elongation project negatively. To evaluate model fitness, we calculated R^2^ to measure the variance captured by the model. Model prediction accuracy was evaluated with Q^2^ using a leave-one-out cross validation [Fig. 6(c)]. A permutation test was used to validate the actual Q^2^ value from a distribution of all possible Q^2^ values obtained when constructing a PLS model from a scrambled data matrix [Figs. S6(e)–S6(g)]. We determined the ideal number of principal components to use such that the Q^2^ and R^2^ are maximized, without overfitting. Overfitting occurs when the R^2^ is high, but the Q^2^ value is low or negative. Two principal components were used for prediction in the MDA-MD-231 model, and three principal components were used for the MDA-MB-468 and combined cell line models.

We then identified how different cell adhesion parameters contribute to prediction of 2D speed, 2D persistence, and 3D invasion using the variance importance parameter (VIP) score for each cell adhesion parameter [Figs. 6(d)–6(f)]. The VIP score reports the amount of variation in 2D speed, 2D persistence, and 3D invasion that is explained by each adhesion parameter. We find that in the MDA-MB-231 and combined cell lines models, all the cell adhesion parameters rank similarly for predicting 2D speed, indicating that all parameters are important for prediction [Figs. 6(d) and S8(d)]. However, in the MDA-MB-468 model, we find that the mean radius, cell area, and min feret diameter, which are measures of cell size, are important for predicting 2D speed [Fig. S7(d)]. Interestingly, in the MDA-MB-231 and combined cell lines models, measures of cell irregularity and elongation are important for predicting 2D persistence and 3D invasion [Figs. 6(e), 6(f), S8(e), and S8(f)]. In the MDA-MB-468 model, we find that measures of cell size and elongation are important for predicting 2D persistence and 3D invasion [Figs. S7(e) and S7(f)]. We find between cell migration speed and persistence, persistence is more important for predicting 3D invasion in both MDA-MB-231 and MDA-MB-468 cells [Fig. 6(f)]. However, in the combined cell line model, cell migration speed is ranked as more important [Fig. S8(f)]. Overall, these models demonstrate the importance of individual shape parameters in predicting ECM-driven migration responses in 2D and 3D.

### Models can be used to accurately predict 2D and 3D ECM-driven responses

We first tested the ability of our three data-driven models to predict the effects of a new ECM protein, within the same cell line: simple correlation, classification, and PLS regression. We chose Matrigel, isolated from the Engelbreth–Holm–Swarm (EHS) mouse sarcoma, which is rich in basement membrane components laminin, Collagen IV, and heparan sulfate proteoglycans. We measured cell adhesion, 2D migration and 3D invasion of MDA-MB-231 and MDA-MB-468 cells in response to Matrigel [Figs. 7(a), 7(b), S9(a), and S9(b)]. We first used the simple correlation models to predict the cell responses to Matrigel [Figs. 7(c), 7(d), S9(c), S9(d), and S10(a)–S10(d)]. The cell area was used to predict 2D cell migration speed and 2D persistence. 3D invasion was predicted from the cell area, cell migration speed, and persistence. Although the cell area predicted Matrigel-driven responses of MDA-MB-231 cells in both 2D and 3D with low percent error, the model fit, R^2^, and cross-validation accuracy, Q^2^, were very low [Figs. 7(c) and 7(d)]. Additionally, the cell area did not accurately predict Matrigel-driven responses of MDA-MB-468 cells [Figs. S9(c) and S9(d)]. This indicates that a simple correlation is not adequate to predict responses to new ECM proteins in 2D and 3D.

Next, we used the classifier models with all parameters to predict the cell responses to Matrigel, since using either all cell adhesion parameters or all cell migration parameters had the best AUROC scores (Figs. 5 and S5). We find that these models have low error in predicting 2D speed, but higher percent error for predicting the magnitude of 3D invasion in Matrigel [Figs. 7(e), 7(f), S9(e), and S9(f)]. Predicting 3D invasion from adhesion shape parameters had lower error and high confidence compared to predictions made from 2D speed and persistence [Fig. 7(f)]. Similar results were obtained for MDA-MB-468 cells [Figs. S9(e) and S9(f)]. These studies suggest that cell adhesion parameters are better able to predict 3D invasion than 2D cell migration.

We used the PLS models to quantitatively predict cell responses to Matrigel in 2D and 3D [Figs. 7(g) and 7(h)]. We found that in general, the PLS models using cell adhesion were able to predict cell responses in 2D as seen from the low percent error and high model confidence (both R^2^ and Q^2^) [Fig. 7(g)]. Next, we looked at how cell adhesion, 2D migration, and both performed in predicting 3D invasion in Matrigel. In MDA-MB-231 cells, we find that cell adhesion and the combination of cell adhesion and 2D migration predicted the effects of Matrigel in 3D accurately, but 2D migration alone did not perform well in predicting responses in 3D [Fig. 7(h)]. We find that only 2D persistence and 3D invasion, but not 2D speed, are well predicted in the MDA-MB-468 model (Figs. S9(g) and S9(h)]. Prediction of Matrigel from the combined model has a larger error, indicating cell line specificity [Figs. S10(e) and S10(F)]. Additionally, using both cell adhesion and 2D migration to predict 3D invasion performed similar to using cell adhesion alone to predict 3D invasion. Overall, cell adhesion can be used to predict ECM-driven responses in both 2D and 3D; however, 2D migration cannot be used to accurately predict 3D invasion.

We then tested the ability of our PLS models to predict the responses of new cell lines, given that this model provided the lowest error and the highest confidence. For these experiments, we used BT-549, another human triple-negative breast cancer cell line. We evaluated the effects of Fibronectin, Tenascin C, and Matrigel on cell adhesion, 2D migration, and 3D invasion of BT-549 cells [Figs. 8(a)–8(d)]. We find that these ECM proteins have distinct effects on the shape, 2D migration, and 3D invasion of the cells, consistent with our previous data. Next, we evaluated how well the MDA-MB-231, MDA-MB-468, and combined models predict the responses of BT-549 to these ECM proteins. We did this with and without training the models with BT-549 data [Fig. 8(e)]. We find that when we trained the original models with BT-549 response to no ECM, the error reduced. Training the models with BT-549 responses to both no ECM and Fibronectin also increases how accurately Tenascin C and Matrigel are predicted, as seen by the lower percent error [Fig. 8(e)]. Interestingly, the MDA-MB-231 model predicts BT-549 response to Tenascin C and Matrigel better than the MDA-MB-468 model. We also find that the models more accurately predict responses to Tenascin C than Matrigel.

## DISCUSSION

Our goal was to identify the relationship between ECM responses in adhesion, 2D migration, and 3D invasion assays to develop strategies to easily predict the effect of novel ECM proteins on 3D cancer cell invasion, which is more relevant to the study of cancer metastasis. 3D invasion assays can be more complex, time consuming, and more challenging for follow up analysis, while 2D migration and adhesion assays are quicker, easy to analyze, and to use with other experimental approaches such as cell sorting, atomic force microscopy, or immunostaining. By evaluating the response of two triple-negative breast cancer cell lines to four ECM proteins known to be upregulated in metastatic breast cancers, we found that there is no linear relationship between metrics used to quantify these three assays. Using the AdaBoost machine learning algorithm, we found that cell adhesion can successfully and accurately classify 2D migration speed and 3D invasion, while 2D migration speed and persistence are unable to classify ECM-driven 3D invasion. ECM proteins have distinct effects on cell adhesion, which is characterized by features that characterize the cell size, irregularity, and elongation. Using data-driven modeling, we find that some shape parameters, such as those that quantify cell elongation and irregularity, are more important for predicting 2D migration and 3D invasion. Finally, we use the correlation, classification, and regression models to predict the effect in 2D and 3D of a new ECM protein. From these data, we see that while the predictions obtained by simple correlation have low percent error, the R^2^ and Q^2^ values are low, suggesting that the confidence in the prediction is low. The predictions obtained by classification are accurate, but do not report an actual value. Finally, the PLS predictions have low percent error, and positive R^2^ and Q^2^ values, which indicate good capture of the variance and high predictive power. We also find that predictions are cell-line specific and that models generated for one cell line cannot be used for another without additional training. Overall, these studies suggest that the shape a cell takes in response to an ECM protein, and not 2D migration speed is more predictive of 3D invasion and our data provide a pipeline to predict the effect of novel ECM proteins in driving invasion and metastasis based on a simple adhesion assay.

In this study, we found that both machine learning classifier models and data-driven PLS models could be used to accurately predict 2D and 3D responses of cells to a new ECM protein. We focused on the AdaBoost classifier, originally developed by Freund and Shapiro in 1995, an approach found on the notion of using a set of weak classifiers and pooling the classification results of such classifiers to produce a provably strong classifier. It is therefore less susceptible to overfitting than other learning algorithms. We acknowledge that our sample size is too low to completely rule out the possibility of over-training, but we were successfully able to classify a new ECM protein for two different cell lines. The limitations of predictions with classifier models could be addressed by using a more quantitative PLS model. We find that our PLS model was able to quantitatively define the relationships between cell adhesion and the 2D and 3D responses of cells by iteratively reducing the dimensionality of the training dataset. With PLS modeling, we are able to extract which cell shape parameters are most strongly connected with cell responses in 2D and 3D, which allows us to generate hypotheses and quantitatively support them.^21^ Nevertheless, the PLS model still had important limitations. When predicting the effects of Tenascin C and Matrigel in the new cell line, the PLS model was more accurate at predicting the effects of Tenascin C. Matrigel is known to be a mixture of several growth factors and ECM proteins, suggesting that its effect on cell migration is more complex. Indeed, we have shown that there can be synergy between growth factors and ECM protein, suggesting that combinations of cues from multiple growth factors and ECM proteins can lead to more complex results.^22^ We also found that the PLS model constructed with MDA-MB-231 cells predicted ECM-driven effects in BT-549 cells better than the PLS model with MDA-MB-468 cells. MDA-MB-231 cells are known to be more mesenchymal, and it has been found that BT-549 cells express characteristics of more mesenchymal cells, as seen by lower surface levels of Integrin B4.^18^ BT-549 was also found to have a similar metastatic potential to MDA-MB-231 cells, suggesting that the two mesenchymal cell lines would respond similarly to different ECM proteins.^19^ Therefore, our model may be best suited for studies within a single cell line and with individual ECM proteins. Future studies will have to address how combinations of ECM cues, with and without other pro-migratory such as growth factors, impact cancer cell invasion.

These studies also shed light on the heterogeneity of responses to ECM proteins in all these assays, particular for the adhesion and migration, where the data are quantified at the single cell level. Some ECM proteins, like Tenascin C and Collagen I, induce more homogeneous responses in terms of cell shape parameters, while others like Fibronectin and Collagen IV have a range of effects (Fig. 2). Previous studies have linked heterogeneity of cell shape to metastatic potential. For example, lower variation in morphology is predictive of cells derived from metastatic sites, but not associated with any particular somatic mutations.^23^ More recently, cell morphology of breast cancer cells was found to predict distinct tumorigenic and metastatic potentials *in vivo* using multiple mouse models of breast cancer.^24^ In addition, the dynamics of breast cancer cell shape heterogeneity can impact response to therapy. Indeed, time series modeling that captures the heterogeneous dynamic cellular responses can improve drug classification and provide insight into mechanisms of drug action.^25^ The mechanisms that govern this heterogeneity in breast cancer remain poorly understood. We have shown that changes in alternative splicing of the actin regulator Mena can impact sensitivity to Fibronectin (FN) gradients *in vivo*. Breast cancer cells that express the Mena^INV^ isoform, which includes a 19 amino acid exon, are more sensitive to FN which increases their metastatic potential.^5,26^ Expression of Mena^INV^ is regulated by the acidity of the local environment,^27^ suggesting that feedback between the tumor microenvironment and the cancer cells themselves is critical in regulating the signaling pathways that will impact cell shape heterogeneity. It will be important to evaluate how the heterogeneity of response to ECM proteins impacts metastasis and response to therapy.

Migration responses in 2D are quantified with two main metrics: cell migration speed and persistence; however, it is not clear what migration response is more relevant to metastasis. Interestingly, in the World Cell Race, speed and persistence correlated for the migration of over 50 cell types on fibronectin coated lines.^28^ In a follow-up study, persistence was found to be robustly coupled to cell migration speed;^29^ however, these studies were performed in epithelial and myeloid cells and are not in response to a given cue. We find no correlation between ECM-driven cell speed and persistence, and that the cell shape is more predictive for persistence than it is of cell speed. Persistence may be more relevant to study in response to a directional cues, such as in the context of haptotaxis or chemotaxis.^3,13^ For example, directed migration of breast cancer cells to gradients of Fibronectin increases directional persistence to promote metastasis, without affecting cell speed.^5^ Therefore, the nature and organization of the cue driving cell migration may play an important role in determining which metric is more predictive of metastasis potential.

Our studies further demonstrate that in the context of ECM responses, the cell shape is predictive of 3D invasion, with ECM-driven effects on 2D speed not predictive of 3D invasion. Cell trajectories in 2D vs 3D environments have also been shown to be different, requiring different methods to predict responses within each context. A persistent-random walk model can be used to accurately model 2D cell migration, while anomalous diffusion models are better suited to describe 3D cell migration in confined environments.^30^ Several studies have reported on the relationship between 2D and 3D migration in the ECM. Fraley *et al.* demonstrated that in Collagen I, changes in 3D cell migration speed did not correlate with changes in 2D cell speed or persistence, but that the extent of focal adhesion protein-mediated protrusion activity is directly correlated with 3D cell speed.^11^ However, this study was done with fibrosarcoma cells embedded in Collagen I, and did not look at these parameters in the context of other ECM proteins. Furthermore, comprehensive analysis of cell motility measurements in 2D and 3D models reveals that only the percent of migrating cells in 2D positively correlates with the cell migration in 3D environments, although these studies did not take into account differences in ECM or biomaterial tissue composition.^31^ Overall, these studies established correlative relationships between these different metrics, but did not evaluate the ability of these quantitative relationships to predict the effects of unknown conditions. Here, we robustly show using different models that we can accurately predict the effect of an ECM protein on 3D invasion based on its effect on cell shape. Finally, these data are similar to what was found for response to growth factors, another pro-migratory cue. Whereas 2D migration properties did not correlate well with 3D behavior across multiple growth factors, Meyer *et al.* found that increased membrane protrusion elicited by growth factor stimulation did relate robustly to enhanced 3D migration properties in several breast cancer cells.^12^ Overall, these studies further support the importance of considering the properties of the cue to best evaluate its role on breast cancer metastasis. Future studies should address the importance of cell speed, persistence, and invasion to metastasis *in vivo*.

## METHODS

There are no experiments on human or animals in this study; therefore, ethics approval is not required.

### ECM substrates

Reagents were purchased from Fisher Scientific (Hampton, NH) or SIGMA (St. Louis, MO) unless otherwise specified. We used the following ECM proteins: Collagen I protein (CB-40236; Fisher Scientific, Hampton, NH), Fibronectin protein (F1141; SIGMA, St. Louis, MO), Tenascin C (R&D systems, 3358TC050), Collagen IV protein (Abcam, ab7536), and Matrigel (growth-factor reduced, Corning, CB-40230C).

### Cell culture

MDA-MB-231, MDA-MB-468, and BT-549 cells were obtained from ATCC (Manassas, VA). MDA-MB-231 and MDA-MB-468 cells were cultured in Dulbecco's Modified Eagle's Medium (DMEM) with 10% serum and Pen-Strep Glutamine and BT-549 were grown in RPMI-1640 + 10%PBS + Insulin (1 *μ*g/ml). Cells were checked every 2 months for the presence of mycoplasma by a polymerase chain reaction (PCR) based method using a Universal Mycoplasma Detection Kit (30–1012K; ATCC, Manassas, VA). Only mycoplasma negative cells were used in this study. Cell lines were made to stably express GFP by lentiviral transduction and labeled as 231-GFP, 468-GFP, or BT-549-green fluorescent protein (GFP).

### Adhesion assay

Plastic-bottomed dishes (Thermo Fisher Nunc, 96 Well Optical-Bottom Plates, 165305) were coated with 20 *μ*g/ml ECM protein for 1 h at 37 °C and then washed with phosphate-buffered saline (PBS). Cells were trypsinized, resuspended in media, and plated on the coated plates at 4000 cells/per well. After 2 h, cells were then fixed for 15 min in 4% paraformaldehyde, then permeabilized with 0.2% TritonX-100, blocked with 3% bovine serum albumin (BSA) and incubated with primary antibodies overnight at 4 °C. Cells were stained with 4′,6-diamidino-2-phenylindole (DAPI) (D1306; Thermo Fisher Scientific, Waltham, MA) and Phalloidin (A12390; Thermo Fisher Scientific, Waltham, MA) along with incubation with fluorescently labeled secondary antibodies at room temperature for 2 h. Imaging was performed using a Keyence BZ-X710 microscope (Keyence, Elmwood park, NJ) and CellProfiler v3.1.8 was used for imaging analysis using a custom pipeline.^32^ Cells were first identified from the nucleus, and the outline of each cell was determined from the cytoplasm staining. Cells at the edge of an image were discarded. 2D adhesion was quantified by 11 parameters: area/cell (number of sq. *μ*m in the cell cytoplasm), aspect ratio (the ratio of the major axis length and the minor axis length of the cell), compactness (mean squared distance of the cell cytoplasm from the centroid divided by the area, where a filled circle has a value of 1, and an irregular shape has a value greater than 1), eccentricity (ratio of the distance between the foci of the ellipse and its major axis length, where a perfect circle has a value of 0, and more elongated cells have a value of 1), extent (proportion of pixels in the bounding box that are also in the cell cytoplasm, where larger values indicate more spread out cell cytoplasm), form factor (calculated as 4 × π × Area/Perimeter^2^, where a perfect circle has a value of 1), max. and min. feret diameter (minimum and maximum distance between two parallel lines that are tangent to the cell cytoplasm edge), mean radius, perimeter, and solidity (proportion of pixels that are in the convex hull that are also in the cell cytoplasm, where a perfect circle has a value of 0). Data are the result of three independent experiments with three technical replicates per experiment.

### 2D migration assay

For 2D migration, 12-well glass-bottomed dishes (MatTek, Ashland, MA) were coated with 20 *μ*g/ml ECM protein for 1 h at 37 °C. ECM was washed off with PBS, and cells were plated at 7500 cells/well on and allowed to adhere. After 1 h, the plate was placed on the microscope and cells were imaged overnight with images acquired every 10 min for 16 h in an environmentally controlled chamber within the Keyence BZ-X710 microscope (Keyence, Elmwood park, NJ). Cells were then tracked using VW-9000 Video Editing/Analysis Software (Keyence, Elmwood park, NJ) and both cell speed and persistence were calculated using a custom MATLAB script vR2018a (MathWorks, Natick, MA). 2D migration was quantified by two parameters: cell migration speed and persistence. Data are the result of three independent experiments with six fields of view per experiment and an average of six cells tracked per field of view.

### Spheroid invasion and migration assay

Cells were seeded in low-attachment plates in media (Corning™ 96 Well Ultra-Low Attachment Treated Spheroid Microplate, 12–456-721), followed by centrifugation at 3000 rpm for 3 min to form spheroids. Spheroids were grown for 3 days after which ECM was added to each well, which included (depending on the condition) Collagen I protein to a 1 mg/ml concentration, ECM protein of interest at 20 *μ*g/ml, 10 mM NaOH, 7.5% 10× DMEM and 50% 1× DMEM. The spheroids in ECM were then spun down at 3000 rpm for 3 min, and the ECM gel left to polymerize for 1 h at 37 °C. After this, a further 50 *μ*l of media added to each well. Following another 5 days of growth, spheroids were imaged as a Z-stack using a Keyence BZ-X710 microscope (Keyence, Elmwood park, NJ) and Z-projection images analyzed using a Hybrid Cell Count feature within the BZ-X Analyzer software v1.3.1.1 (Keyence, Elmwood park, NJ). 3D invasion was quantified by one parameter: increase in surface area on day 5 of ECM relative to day 1. Data are the result of three independent experiments with six technical replicates per experiment.

### Clustering analyses

SPRING plots were constructed from single cell adhesion quantification using the methods described in Weinreb *et al.*^20^ For large scale quantification of cell adhesion on different ECM substrates, each profile was averaged and mean centered. The ECM factors and adhesion metrics were clustered by rank correlation and mean linkage, using the seaborn package for Python. Cell adhesion shape metrics were compared by calculating the Spearman correlation between each pair of metrics.

### Machine learning classification

ECM-driven effects on 2D migration and 3D invasion were classified as either low or high (see Fig. 5). For classification of 2D migration in MDA-MB-231 and MDA-MB-468 cells, ECM substrates that caused a mean cell migration speed of above 0.5 *μ*m/min were classified as high. For classification of 3D invasion in MDA-MB-231 cells, ECM substrates that caused a mean fold change in spheroid area of above ten were classified as high. For classification of 3D invasion in MDA-MB-468 cells, ECM substrates that caused a mean fold change in the spheroid area of above eight were classified as high. The machine learning algorithm AdaBoost in the sk learn package was used. We first verify in a grid search of the two hyperparameters (learning rate and number of estimators) that AdaBoost is quite robust to different settings. Since many combinations of hyperparameters gave an average AUROC value of 0.75 or higher on the training set (Fig. S5), we decided to set the hyperparameters to the recommended defaults in the sk learn package (learning rate of 1, number of estimators at 50). AUROC area under the curve was used to assess the accuracy of the classifiers. The optimized models were tested using a new unknown ECM protein. All machine learning classifications were performed using Python.

### Principal component analysis and partial least-squares regression

Principal component analysis and partial least squares regression were performed as described previously.^21^ Model fitness was evaluated using R^2^, which reports the variance captured by the model. Model prediction accuracy was first evaluated using a leave-one-out cross validation metric, Q^2^, previously described.^33^ A permutation test was used to validate the actual Q^2^ value from a distribution of all possible Q^2^ values obtained when constructing a PLS model from a scrambled data matrix. The model was tested with a new condition (ECM protein or cell line), and this prediction was evaluated using percent error. VIP scores were calculated from reference.^34^ Adhesion parameters with VIP scores above 1 were considered as important cell adhesion parameters for prediction. All data were scaled to nondimensionalize the different metrics. PCA was performed using Python, and the PLS model was implemented using Matlab.

### Statistical analysis

GraphPad Prism v7.04 was used for generation of graphs and statistical analysis. All statistical comparisons were done between no ECM condition and each ECM condition individually. A one-way analysis of variance (ANOVA) was used with a corrected p-value of ≤0.05 considered significant.

## SUPPLEMENTARY MATERIAL

See the supplementary material for additional figures (Figs. S1–S10), which provide data for the MDA-MB-468 cell line, model optimization data, and predictions for MDA-MB-468 cells from the single cell line dataset and from the dual cell line dataset.

## AUTHOR'S CONTRIBUTIONS

J.P.B. and A.W. contributed equally to this work.
